# Path-Integral Calculation of the Second Dielectric and Refractivity Virial Coefficients of Helium, Neon, and Argon

**DOI:** 10.6028/jres.125.022

**Published:** 2020-08-06

**Authors:** Giovanni Garberoglio, Allan H. Harvey

**Affiliations:** 1 European Centre for Theoretical Studies in Nuclear Physics and Related Areas (FBK-ECT*) and Trento Institute for Fundamental Physics and Applications (TIFPA-INFN), Trento, I-38123, Italy; 2 Applied Chemicals and Materials Division, National Institute of Standards and Technology, Boulder, CO 80305, USA

**Keywords:** argon, dielectric virials, helium, neon, path-integral Monte Carlo, pressure, refractivity virials, thermometry

## Abstract

We present a method to calculate dielectric and refractivity virial coefficients using the path-integral Monte Carlo formulation of quantum statistical mechanics and validate it by comparing our results with equivalent calculations in the literature and with more traditional quantum calculations based on wavefunctions. We use state-of-the-art pair potentials and polarizabilities to calculate the second dielectric and refractivity virial coefficients of helium (both ^3^He and ^4^He), neon (both ^20^Ne and ^22^Ne), and argon. Our calculations extend to temperatures as low as 1 K for helium, 4 K for neon, and 50 K for argon. We estimate the contributions to the uncertainty of the calculated dielectric virial coefficients for helium and argon, finding that the uncertainty of the pair polarizability is by far the greatest contribution. Agreement with the limited experimental data available is generally good, but our results have smaller uncertainties, especially for helium. Our approach can be generalized in a straightforward manner to higher-order coefficients

## 1 Introduction

Capacitance-based measurements that employ noble gases have several applications (and potential applications) in metrology. Dielectric-constant gas thermometry [1] has been used for primary temperature measurement [2], and it provided a key input for the recent redefinition of the Boltzmann constant [3, 4]. A proposal for basing pressure standards on capacitance measurements was first made over 20 years ago [5], and primary pressure measurement up to 7 MPa has recently been demonstrated based on measuring the static dielectric constant (relative permittivity) of helium [6]. While most such metrology is performed with helium, neon and argon may also be used because their larger polarizabilities increase the sensitivity of apparatus [6, 7].

Measurements of the refractive index of noble gases also have applications in metrology. A recent review article [7] described refractive-index gas thermometry, and work is ongoing to develop a primary pressure standard based on the refractivity of helium [8, 9].

In the applications based on capacitance, the static dielectric constant *ε* is related to the molar density *ρ* by the virial expansion of the Clausius–Mossotti function:

$\frac{\varepsilon -1}{\varepsilon +2}$ =*ρ*(*A*_ε_+*B*_ε_*ρ*+*C*_ε_*ρ*^2^+...)=*ρA*_ε_(1+*bρ*+*cρ*^2^+...), (1)

where *A_ε_* is proportional to the mean static polarizability of the isolated molecule. The term “second dielectric virial coefficient” is used in the literature sometimes for *B_ε_* and sometimes for *b*; the two are related by *B_ε_* = *A_ε_ b*. Similarly, the third dielectric virial coefficient is sometimes defined as *c* and sometimes as *C_ε_* = *A_ε_ c*; we will use *B_ε_* and *C_ε_* in this work. For a given species, these virial coefficients are functions only of temperature. *B_ε_* depends on interactions between two molecules, *C_ε_* depends on interactions among three molecules, and so forth. For nonpolar species such as noble gases, the *A_ε_* term dominates in Eq. (1), and the virial terms are only significant for precise work.

For the refractive index *n*, a parallel expansion exists for the Lorentz–Lorenz function:

$\frac{n²-1}{n²+2}=\rho$(*A*_R_+*B*_R_*ρ*+*C*_R_*ρ*^2^+...), (2)

where *A*_R_, *B*_R_, and *C*_R_ are analogous refractivity virial coefficients that depend on temperature and frequency. In the limit of zero frequency, the refractivity virial coefficients are equal to their dielectric counterparts (with the exception of a small term involving the magnetic permeability, which is known approximately for *A*_R_ and is negligible for the higher coefficients [10]), but at finite frequencies a correction for dispersion is needed. The dispersion correction at optical frequencies is quite small for the noble gases because there is negligible absorption between optical frequencies and the static limit. The dispersion correction for *B*_R_ is given to lowest order as

$B_{R}=B\varepsilon +\omega ²B_{R}^{\left(2\right)}$, (3)

where *ω* is the angular frequency. The expansion coefficient $B_{R}^{\left(2\right)}$ depends on an interaction-induced Cauchy moment, ∆*S*(−4), corresponding to the pair interaction, as described by Koch *et al.* [11].

In parallel with Eq. (1) and Eq. (2), dielectric and refractivity measurements in metrology employ the more familiar thermodynamic virial expansion for the pressure *p*, which is a systematic series of corrections to the ideal-gas law:

$\frac{\rho }{\rho RT}=1+B\rho +C\rho ²+...$(4)

where *T* is the absolute temperature and *R* is the molar gas constant. The second virial coefficient *B* depends only on the interaction between two molecules, the third virial coefficient *C* includes three-body interactions, *etc.*; these coefficients are only functions of temperature for a given species. Equations (1) and (4) are combined to fully describe the deviation of gas-phase dielectric-constant measurements from ideal-gas behavior; see Refs. [1] and [6] for details. An analogous combination of Eq. (2) and Eq. (4) is performed to describe gas-phase refractivity measurements [7].

For helium, it is possible to construct capacitance-based and refractivity-based primary standards of temperature and pressure due to the availability of highly accurate *ab initio* calculations. The static polarizability of an isolated helium atom (and therefore *A_ε_*) is now known with a relative standard uncertainty of 0.1 × 10^−6^ (0.1 ppm) [10, 12, 13], and the frequency-dependent polarizability (and therefore *A*_R_) is known at optical frequencies with a similarly small uncertainty [10]. The availability of a highly accurate *ab initio* pair potential, and a fairly accurate three-body potential, enables accurate calculation of *B* [14] and *C* [15] as functions of temperature.

The dielectric and refractivity virial coefficients beyond the first, however, are much less well known, both from experiment and theory, and they can contribute significantly to metrological uncertainty budgets [6, 7]. We will defer discussion of the experimental situation until later sections; in the following we discuss theoretical calculation of *B_ε_* and *B*_R_ for noble gases.

The theoretical formulae for the dielectric virial coefficients were derived by Moszynski *et al.* [16], who showed that the dielectric virial of order *n* requires knowledge of the interaction potential of *n* molecules, *V_n_*, as well as the interaction-induced polarizability of the same *n* particles, ***α****_n_*, which, in general, is a 3 × 3 matrix. All of these quantities can in principle be calculated using *ab initio* electronic structure methods.

Since the computational effort required to obtain potentials and polarizabilities is quite significant, this task has been undertaken up to now only for a few monatomic gases and limited to the second dielectric virial coefficient, *B_ε_* (*T*), which is given by 

$B\varepsilon (T)=\frac{2\pi N_{A}^{2}}{3\gamma }\Lambda_{m}^{6}\int d³r_{1}^{\left(1\right)}d³r_{2}^{\left(1\right)}\left[\langle r_{1}^{\left(1\right)},r_{2}^{\left(1\right)}|\Delta \alpha ₂(r)e^{-\beta H}|r_{1}^{\left(1\right)},r_{2}^{\left(1\right)}\rangle +\frac{{\left(-1\right)}^{2I}}{2I+1}\langle r_{1}^{\left(1\right)},r_{2}^{\left(1\right)}|\Delta \alpha_{₂}(r)e^{-\beta H}|r_{2}^{\left(1\right)},r_{1}^{\left(1\right)}\rangle \right]$, (5)

where **$r_{1}^{\left(1\right)}$**and $r_{2}^{\left(1\right)}$ denote the positions of the two atoms (and the superscript ^(1)^ is introduced for later convenience), $r=|r_{2}^{\left(1\right)}-r_{1}^{\left(1\right)}|$is their distance, *H*=*K*_1_+*K*_2_+*V*_2_(*r*) is the Hamiltonian describing the interaction between two atoms (with *K_j_* being the kinetic energy operator for atom *j*), ∆*α*_2_(*r*) is one third of the trace of the interaction-induced pair polarizability ***α***_2_, *k*_B_ is the Boltzmann constant (we use this symbol to avoid confusion with the use of *k* for wavevectors in Sec. 2), *β* = (*k*_B_*T*)^−1^, and *N*_A_ is the Avogadro constant. In Eq. (5), γ is the volume of the box enclosing the two atoms, with the understanding that the actual value is obtained in the γ → ∞ limit, and $\Lambda m=h/\sqrt{2\pi mk_{B}T}$ is the thermal de Broglie wavelength of an atom whose mass is denoted by *m*. The first term in square brackets in Eq. (5) is called the *direct* term, whereas the second term takes into account quantum statistics; bosons have integer nuclear spin *I*, whereas fermions have half-integer nuclear spin.

Values of *B_ε_* (*T*) for ^4^He were computed by Moszynski and collaborators using an empirical pair potential together with a first-principles calculation of ∆*α*_2_(*r*) [16, 17]. Quantum effects were taken into account either with a semiclassical expansion up to *O*(*h* ^4^), or with a fully quantum approach described in Sec. 2 below.

Subsequently, Rizzo *et al.* recalculated *B_ε_* (*T*) for helium using a fully *ab initio* pair potential and polarizabilities, obtained at the FCI (Full Configuration Interaction) level with large basis sets [18]. These new calculations showed a significant difference in the values of the second dielectric virial coefficient, especially at the lowest temperature, and this disagreement was attributed to improved accuracy in the interaction-induced polarizability ∆*α*_2_. 

Recently, further advances in *ab initio* calculations have provided a more accurate pair potential for the helium dimer [19] as well as an improved interaction-induced polarizability [20]. Additionally, the developers of these values provided accuracy estimates, enabling estimation of the uncertainty in the calculated dielectric virial coefficient. In previous works, the overall uncertainty of *B_ε_* (*T*) was never estimated in a rigorous way; the first paper to present a rigorous uncertainty analysis, albeit in a semiclassical framework, appeared while this manuscript was being finalized [21].

*Ab initio* pair potentials and polarizabilities are also available for neon and argon. In the case of neon, we used the pair potential calculated by Hellmann *et al.* [22] together with the pair polarizability by Hättig *et al.* [23]. Much effort has been devoted to argon, given its importance in many metrological applications. We used the *ab initio* pair potential of Patkowski and Szalewicz [24], who also estimated its uncertainty. In addition, we used the pair polarizability of Vogel *et al.* [25].

In this study, we developed an alternative way of calculating *B_ε_* (*T*) using the path-integral Monte Carlo (PIMC) approach. This method has been proven to provide accurate fully quantum results for the density virial coefficients of atoms and molecules [26–28], and it can be used to calculate virials of any order in a straightforward manner [15, 29, 30]. Following Cencek *et al.* [20], we evaluated the uncertainty of *B_ε_* (*T*) as a function of the uncertainties of *V*_2_(*r*) and ∆*α*_2_(*r*).

We also made use of our values of *B_ε_* to calculate the second refractivity virial coefficient *B*_R_ from Eq. (3). This requires computing $B_{R}^{\left(2\right)}$, for which the expression [11] is analogous to Eq. (5), provided that the Cauchy moment, ∆*S*(−4), is used instead of ∆*α*_2_. These Cauchy moments are given as a function of radial distance by Hättig *et al.* [31] for helium, Hättig *et al.* [23] for neon, and Fernández *et al.* [32] for argon. In the case of argon, only tabular values were given, so we fitted those values to the same functional form used for neon in Ref. [23].

## 2 The Wavefunction Approach

The usual way [17, 18] to calculate *B_ε_* (*T*) starting from Eq. (5) is to first introduce the center of mass and relative coordinates, that is, **R** = ( $r_{1}^{(1)}+r_{2}^{(1)}$)*/*2 and **r** = $r_{2}^{(1)}-r_{1}^{(1)}$, respectively. Using these variables, the Hamiltonian *H* separates into a trivial center-of-mass component and a part involving only the relative coordinates. One can then insert a completeness relation of the form

$1=\sum_{l}(2l+1)\left(\sum_{n}|\psi_{nl}\rangle \langle \psi_{nl}|+\int_{0}^{\infty }dk|\psi_{kl}\rangle \langle \psi_{kl}|\right),$ (6)

where *l* denotes the angular momentum, |*ψ_nl_*〉 are the bound states of the pair potential (for which energies will be denoted by *E_nl_*), and |*ψ_kl_*〉 are the solutions of the radial Schrödinger equation with energy *E_k_* = *h* ^2^*k*^2^*/*(2*µ*), where *µ* is the reduced mass of the atomic pair. Apart from the usual condition 〈*ψ_nl_*|*ψ_nʹ__lʹ_*〉 = 1, Eq. (6) implies that the normalization of the radial part of the continuum wavefunctions |*ψ_kl_*〉 is

〈*ψ_kl_*|*ψ_k’l’_*〉 = *δ* (*k* − *kʹ)* *δ_llʹ_,* (7)

which is in turn a condition on the amplitude of their oscillation at distances *r* such that *V* (*r*) is negligible. As is well known, in this region, one has the asymptotic expansion [33]

*ψ_kl_*(*r*) = *A* (cos *δ_l_*(*k*) *j_l_*(*kr*) − sin *δ_l_*(*k*)*y_l_*(*kr*)) , (8)

where *j_l_*(*kr*) and *y_l_*(*kr*) are the spherical Bessel functions (that is, the radial eigenfunctions of the free particle motion), and *δ_l_*(*k*) are the phase shifts. Equation (7) implies that

*A* = $\sqrt{\frac{2}{\pi }k.}$(9) 

Substituting Eq. (6) in Eq. (5), one obtains the expression [16]

$B\varepsilon (T)=\frac{2\pi \Lambda_{\mu }^{3}N_{A}^{2}}{3}\sum_{l}\left(1+\frac{{\left(-1\right)}^{l+2I}}{2I+1}\right)(2l+1)\left[\sum_{n}e^{-\beta {Ε}_{\mathrm{nl}}}\int_{0}^{\infty }r^{2}dr\Delta \alpha_{2}(r)|\psi_{\mathrm{nl}}(r){|}^{2}+\int_{0}^{\infty }dke^{-\beta {Ε}_{k}}\int_{0}^{\infty }r^{2}dr\Delta \alpha_{2}(r)|\psi_{\mathrm{kl}}(r){|}^{2}\right],$(10)

for which evaluation is straightforward. The bound states can be obtained by numerical diagonalization of the Hamiltonian, whereas the continuum states can be obtained by Numerov integration with a step size *d*.

In this last case, the initial conditions can be set as *ψ_kl_*(*r*_0_) = 0 and *ψ_kl_*(*r*_0_ + *d*) = 1, starting from a point where *V* (*r*_0_)˃˃ *E_kl_* and integrating forwards. With this choice of boundary condition, *ψ_kl_*(*r*) tends to diverge, so it is convenient to renormalize its values as the integration proceeds and finally impose the condition of Eq. (9) in the region where *V* (*r*) ∼ 0. Convergence in Eq. (10) depends on the choice of the step size *d* for the numerical evaluation of the wavefunctions (for either bound or scattering states), as well as the number of angular momenta and the number of wavevectors *k* used to perform the integration. In general, the number of angular momenta increases with the cutoff on the wavevector, which in turn depends on the temperature *T* . In the case of helium, we found it convenient to follow the indications in Ref. [34].

### 2.1 Alternative Diagonalization Approach

The procedure outlined above works well for helium and neon, and we obtained good agreement with the results of the path-integral method described below. In the case of argon, however, we noticed a systematic difference between the two approaches. As noted in Ref. [18], one has to be careful in dealing with the many resonances that are present in this system, which result in sharp peaks appearing in the function to be integrated with respect to the wavevector in Eq. (10). However, even inserting more than 10 000 wavevectors in the resonance region, and thus obtaining well-resolved peaks, we noticed a persistent deviation between this method and the classical or the path-integral calculations (on the order of 10% at *T* = 100 K), for which we were unable to pinpoint its origin. We surmise it to be due to a zero-energy resonant state in the *l* = 0 angular momentum sector; its presence is apparent by the fact that the *s*-wave phase shift tends to an odd integer multiple of *π/*2 for *k* → 0 [35], but this exotic state (for which the wavefunction has a formal exponential divergence at large distances) is not taken into account in Eq. (6).

We decided to resort to an alternative method to calculate Eq. (5) by noting that it is expressed in the form of a trace. We then evaluated it by putting the system inside a large sphere of radius *R* and diagonalizing the Hamiltonian. Denoting the eigenstates of *H* in this geometry by |*E_l,n_*〉, the expression of the second dielectric virial coefficient becomes simply

$B\varepsilon (T)=\frac{2\pi N_{A}^{2}}{3}\sum_{l}\left(1+\frac{{\left(-1\right)}^{l+2I}}{2I+1}\right)(2l+1)\sum_{n}e^{-\beta E_{\mathrm{l,n}}}$〈*E*_*l*_,_*n*_|*Δα*_2_(*r*)|*E*_*l*_,_*n*_〉. (11)

We found that using *R* = 4 nm, with a discretization step of *d* = 10^−3^ nm and including 1000 angular momenta, was sufficient to achieve well-converged results. In this way, we were able to confirm the values of *B_ε_* (*T*) obtained for helium and neon using both the approach outlined in the previous sections and the PIMC approach described below, as well as cure the discrepancy observed in the case of argon. The fact that we were able to obtain very similar results (to four significant figures) for the second dielectric virial coefficient using two independent methods that take into account quantum effects with no uncontrolled approximations bolsters our confidence in the accuracy of the values of *B_ε_* (*T*) reported in this paper.

### 2.2 Classical Limit

The classical (large-temperature) limit of Eq. (5) can be obtained by assuming that [*K_i_,V* ] = 0. In this case, the term proportional to (2*I* + 1)^−1^ tends to zero (which is tantamount to saying that both bosonic and fermionic quantum statistics tend to classical statistics in the classical limit), and the matrix elements of the kinetic energy operators are constant, that is,

$\langle r_{i}^{\left(1\right)}|e^{-\beta K_{i}}|r_{i}^{\left(1\right)}\rangle =\frac{1}{\Lambda_{m}^{3}}$(12)

so that the second dielectric virial coefficient becomes

$B_{\varepsilon }^{\mathrm{cl}}(T)=\frac{8\pi^{3}N_{Α}^{2}}{3}\int r^{2}\Delta \alpha_{2}(r)e^{-\beta V(r)}dr,$(13)

which matches the classical result derived by Buckingham [36, 37].

## 3 The Path-Integral Approach

In this section, we show how to evaluate Eq. (5) using the path-integral approach to quantum statistical mechanics [38]. In this way, we will derive a numerical approach equivalent to the wavefunction-based method described in Sec. 2, which will be shown to be more computationally efficient. We also plan to extend it to higher-order coefficients.

In the path-integral approach, one rewrites Eq. (5) using a Trotter expansion for the exponential. That is,

$e^{-\beta Η}=\prod_{i=1}^{P}e^{-\beta Η/P}≃\prod_{i=1}^{P}e^{-\beta K_{1}/P}e^{-\beta K_{2}/P}e^{-\beta V_{2}/P}$,(14)

where the last equality is exact in the limit *P* → ∞. Additionally, we insert in Eq. (5) *P* − 1 completeness relations of the form

$1=\prod_{i=2}^{P}\int d^{3}r_{1}^{\left(i\right)}d^{3}r_{2}^{\left(i\right)}|r_{1}^{\left(i\right)},r_{2}^{\left(i\right)}\rangle \langle r_{1}^{\left(i\right)},r_{2}^{\left(i\right)}|,$(15)

thus expressing the second dielectric virial coefficient as a large multidimensional integral.

Acting on the position eigenstates, all the exponentials involving the potential become numbers so that they can be collected, resulting in a term of the form

$\exp\left[-\frac{\beta }{P}\sum_{i=1}^{P}V_{2}\left(\left|r_{2}^{\left(i\right)}-r_{1}^{\left(i\right)}\right|\right)\right],$ (16)

whereas the matrix elements of the exponential of the kinetic energy operators *K*_1_ and *K*_2_ can be evaluated exactly [15, 26]. In the first (direct) term in Eq. (5), these matrix elements, together with the $\Lambda_{m}^{6}$ factor, result in appearance of the so-called *P*-bead ring-polymer probability distribution for each of the two atoms [39]. This quantity corresponding to atom *j* is given by

$\Pi_{j}=\Lambda_{m}^{3}(\frac{P^{3/2}}{\Lambda_{m}^{3}}{)}^{P}\exp\left[\frac{\pi P}{\Lambda_{m}^{2}}\sum_{i=1}^{P}|r_{j}^{\left(i+1\right)}-r_{j}^{\left(i\right)}{|}^{2}\right],$(17)

where we have denoted $r_{j}^{(P+1)}=r_{j}^{(1)}$. Since we arbitrarily assigned the indices in the superscripts, the integral providing *B_ε_* (*T*) is invariant when we substitute in the polarizability

$\Delta \alpha_{2}\left(\left|r_{2}^{\left(1\right)}-r_{1}^{\left(1\right)}\right|\right)\to \Delta \alpha_{2}\left(\left|r_{2}^{\left(i\right)}-r_{1}^{\left(i\right)}\right|\right)$(18)

for each value of the index *i*, and hence we can replace $\Delta \alpha_{2}\left(\left|r_{2}^{\left(1\right)}-r_{1}^{\left(1\right)}\right|\right)$with

$\bar{\Delta \alpha_{2}}\equiv \frac{1}{P}\sum_{i=1}^{P}\Delta \alpha_{2}\left(\left|r_{2}^{\left(i\right)}-r_{1}^{\left(i\right)}\right|\right).$(19)

Finally, the direct term can then be written as

**Fig.1:**
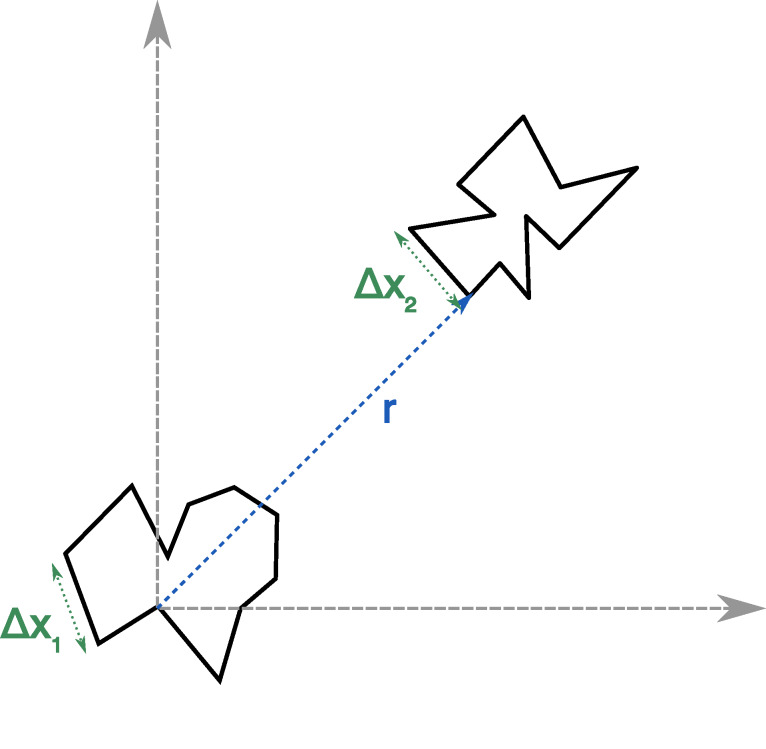
Representation of the coordinates of the ring polymers of the direct term in Eq. (5). Only two representatives of the set of coordinates $\Delta x_{j}^{\left(i\right)}$ are shown for *j* = 1 and *j* = 2.

${Β}_{\varepsilon }^{dir}(T)=\frac{2\pi N_{A}^{2}}{3\gamma }\int \prod_{j=1}^{2}\prod_{i=1}^{P}d^{3}r_{j}^{\left(i\right)}\bar{\Delta \alpha_{2}}\Pi_{1}\Pi_{2}\exp\left[-\frac{\beta }{P}\sum_{i=1}^{P}V_{2}\left(\left|r_{2}^{\left(i\right)}-r_{1}^{\left(i\right)}\right|\right)\right]$ (20)

which can be further simplified using the following considerations. First of all, the integrand is invariant under a translation of all the coordinates, and so it is proportional to the volume γ (which cancels the volume in the denominator), and we can fix one of the coordinates ($r_{1}^{\left(1\right)}$, say) at the origin of the coordinate system. Among the remaining 2*P* − 1 coordinates, 2(*P* − 1) are relative coordinates appearing in the functions Π *_j_*, that is, the coordinates $\Delta x_{j}^{\left(i\right)}=r_{j}^{\left(i+1\right)}-r_{j}^{\left(i\right)}$ for *i* = 2, *. . ., P*, and the last coordinate $r_{2}^{\left(1\right)}$ can be renamed as **r**. A pictorial representation of these coordinates is provided in Fig. 1.

Defining the average pair potential, analogous to $\bar{\Delta \alpha ₂}$ in Eq. (19), as

$\bar{V_{2}}=\frac{1}{p}\sum_{i=1}^{P}V_{2}\left(\left|r_{2}^{\left(i\right)}-r_{1}^{\left(i\right)}\right|\right)$, (21)

and denoting by 〈· · ·〉 the integral over the $\Delta x_{j}^{\left(i\right)}$ variables weighted with Π_1_Π_2_, the path-integral expression for the direct contribution to *B_ε_* (*T*) becomes

$B_{\varepsilon }^{dir}(T)=\frac{2\pi N_{A}^{2}}{3}\int d^{3}r\langle \bar{\Delta \alpha ₂}\exp(-\beta \bar{V₂})\rangle .$ (22)

At high temperatures, the probability distributions Π *_j_* shrink to a point, and hence we recover the classical expression given by Eq. (13) for *B_ε_*, since

$\bar{\Delta \alpha ₂}$→Δα₂

$\bar{V_{2}}$ → *V*_2_.

We can apply the same procedure to evaluate the exchange term. In this case, however, the presence of the exchanged coordinates implies that $r_{1}^{(P\mathrm{+1)}}=r_{2}^{\left(1\right)}$ and $r_{2}^{(P\mathrm{+1)}}=r_{1}^{\left(1\right)}$ . It is then useful to define 2*P *coordinates **y**_*i*_ as **y**_*i*_= $r_{1}^{\left(i\right)}$ for *i*=1,…,*P *and **y**_*i*_= $r_{2}^{(i-P)}$ for *i*=*P*+1,…,2*P*, so that the exchange condition implies that **y**_2*P*+1_=**y**_1_. Analogous to the case of the direct term, we can then define ∆**y***_i_* = **y***_i_*_+1_ − **y***_i_*, and we notice that the product Π_1_Π_2_ can be written in these coordinates as

$\prod ₁\prod ₂=\Lambda_{m}^{6}{\left(\frac{P^{3/2}}{\Lambda_{m}^{3}}\right)}^{2P}\exp\left[-\frac{\pi P}{\Lambda_{m}^{2}}\sum_{i=1}^{2P}|\Delta yᵢ{|}^{2}\right].$(23)

Defining now $\lambda =\sqrt{2}\Lambda$_m_, Eq. (23) can be written as

$\prod ₁\prod ₂=\frac{\lambda^{3}}{8}\lambda^{3}{\left(\frac{{\left(2P\right)}^{3/2}}{\lambda^{3}}\right)}^{2P}\exp\left[\frac{\pi 2P}{\lambda^{2}}\sum_{i=1}^{2P}|\Delta yᵢ{|}^{2}\right]\equiv \frac{\lambda^{3}}{8}\prod ,$(24)

where we recognize the probability distribution Π of a ring polymer with 2*P* beads corresponding to a particle of mass *µ* = *m/*2; see Eq. (17). Analogous to what happened in the case of the direct term, the multidimensional integral providing $B_{e}^{ex}$(*T*) is invariant under an overall translation of the coordinates **y***_i_*, and so it is proportional to the volume γ, provided that we fix one of the coordinates (**y**_1_, say) at the origin of the coordinate system. Defining, analogous to Eq. (19) and Eq. (21),

$\tilde{\Delta \alpha ₂}=\frac{1}{P}\sum_{i=1}^{P}\Delta \alpha ₂$(|y_*i*+*P*_-y_*i*_|)(25)

$\tilde{V₂}=\frac{1}{P}\sum_{i=1}^{P}V₂$(|y_*i*+*P*_-y_*i*_|)(26)

the exchange term can be written as

$B_{\varepsilon }^{ex}(T)=\frac{{\left(-1\right)}^{2I}}{2I+1}\frac{\pi \lambda^{3}N_{Α}^{2}}{12}\langle \tilde{\Delta \alpha ₂}\exp(-\beta \tilde{V₂})\rangle ,$(27)

where the average is taken over the configurations of a closed ring polymer having 2*P* beads distributed according to the function Π defined in Eq. (24). Since the size of the ring polymer described by the distribution Π shrinks to a point at high temperatures, the contribution of the exchange term, Eq. (27), to the dielectric virial is strongly suppressed by the exponential of $\tilde{V₂}$ , which will sample configurations in the repulsive region of the interaction potential. It is only when the size of the ring polymer (which is of the order of *λ*) reaches the order of the repulsive region that we would expect the exchange term to contribute significantly to the dielectric virial. Using the expression of the de Broglie wavelength and the fact that the “size” of a helium atom is on the order of 0.25 nm, exchange effects are expected to become apparent at temperatures lower than about 20 K.

### 3.1 Computational Details

The second dielectric virial coefficient of atomic species is then given by summing the contributions of Eq. (22) and Eq. (27). The radial integration in Eq. (22) was performed using the VEGAS Monte Carlo algorithm, whereas the averages over the ring-polymer configuration were performed using the analytical formula developed by Levy [40, 41] to generate configurations and using at least eight independent samples for each value of the radial coordinate. The exchange contribution depends only on the ring-polymer configurations, and in that case we used 10^5^ samples to evaluate this contribution. The path-integral expressions are exact in the *P* → ∞ limit, although in actual applications a large enough value of *P* suffices. We found convergence in our results using a value of *P* depending on temperature according to *P* = int(2000 K*/T* + 7) in the case of ^3^He, *P* = int(1600 K*/T* + 7) for ^4^He, *P* = int(800 K*/T* + 4) for neon, and *P* = int(300 K*/T* + 4) for argon, where int(*x*) denotes the nearest integer to *x*.

The calculation of the eigenstates |*E_l,n_*) and the corresponding matrix elements of the polarizability needed in Eq. (11) required roughly 32 hours on a modern 2.5 GHz processor for each atomic species considered. After this preliminary calculation, the evaluation of *B_ε_* (*T*) at each temperature takes only a few seconds. On the other hand, the path-integral evaluation of the second dielectric virial coefficient takes a computational time roughly proportional to the Trotter index *P* needed to reach convergence at a given temperature. The most demanding computation for this paper—the calculation for ^3^He at 1 K—took roughly 5 minutes on the same hardware. Therefore, once the programs were properly debugged, all the path-integral calculations presented here could be performed during a lunch break.

In general, the results obtained using PIMC are in exceptional agreement with the results obtained using the more traditional wavefunction-based methods, usually to at least four significant figures. In the following tables, we report the values of *B_ε_* (*T*) and $B_{R}^{\left(2\right)}$ (*T*) obtained with PIMC. We also report, as Supplemental Material, the values of the same coefficients at temperature intervals of 1 K, in the range 1–2000 K for the helium isotopes, 4–2000 K for the neon isotopes, and 50–2000 K for ^40^Ar. In the Supplemental Material, the virials were generally evaluated using the wavefunction-based method, which is more efficient for this kind of systematic calculation in the case of substantial quantum effects, except for argon, because its almost classical nature makes the PIMC approach more suitable.

## 4 Types of Experimental Data

Since we will be comparing our calculated values with experimental *B_ε_* and *B*_R_ in subsequent sections, we briefly review the ways in which their values can be derived from experiments.

The most straightforward method would be direct application of Eq. (1) or Eq. (2), measuring the static dielectric constant *ε* or refractive index *n* of a gas at a fixed temperature over a range of pressures [and therefore a range of densities, where density can be derived from temperature and pressure with a reference equation of state or with Eq. (4) if the density virial coefficients are well known]. In practice, however, while these direct experiments can yield highly accurate values of *A_ε_* and *A*_R_, they are not able to determine the slope in Eq. (1) and Eq. (2) well enough to determine *B_ε_* and *B*_R_ accurately for nonpolar species such as those considered here.

For nonpolar species, the most reliable experimental values of *B_ε_* have been obtained from expansion measurements that reduce systematic errors by employing two or more near-identical vessels, following the general procedure outlined by Orcutt and Cole [42]. All of our comparisons below will be to data from this method unless otherwise stated. Similarly, most reported values of *B*_R_ arise from differential expansion measurements.

Some experimenters report the quantity *b*, which is *B_ε_ /A_ε_* . To convert *b* to *B_ε_*, we used the best current value of *A_ε_* for each gas. For ^4^He, *A_ε_* has been calculated from *ab initio* quantum mechanics more accurately than it can be measured [10, 12, 13]; the value is 0.51725408(5) cm^3^ mol^−1^. For neon and argon, the most accurate values come from Gaiser and Fellmuth [43], who reported 0.9947114(24) cm^3^ mol^−1^ for neon and 4.140686(10) cm^3^ mol^−1^ for argon.

Dielectric-constant gas thermometry can yield accurate values of the quantity (*B* − *b*), where *B* is the density-series second virial coefficient in Eq. (4). When highly accurate values of *B* are known independently (as is the case for helium), the resulting values of *b* can be converted to *B_ε_* as described in the preceding paragraph. For neon and argon, there is enough uncertainty in the values of *B* that this approach produces *B_ε_* with a relatively large uncertainty.

## 5 Results for Helium

### 5.1 Comparison with Other Methods for ***B_ε_***

**Fig. 2:**
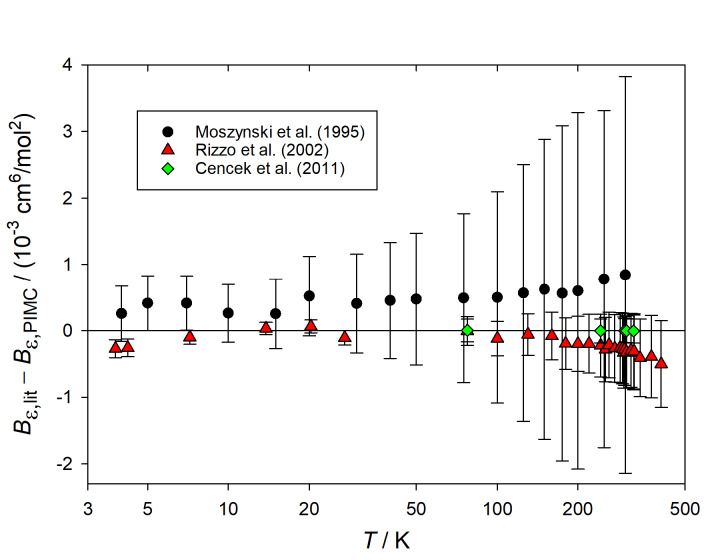
Difference between the values of *B_ε_* (*T*) reported in the literature (*B_ε,_*_lit_) and those calculated with the path-integral method developed in this paper (*B_ε,_*_PIMC_). The error bars indicate the combination of our statistical uncertainty and the uncertainty claimed in the literature source. Circles: Moszynski *et al.* (1995) [16, 17]; triangles: Rizzo *et al.* (2002) [18], assuming 1% uncertainty in their calculated values; diamonds: Cencek *et al.* (2011) [20], using classical values. In all cases, *B_ε,_*_PIMC_ was computed using the pair potential and polarizabilities used in the original literature source. Note the logarithmic temperature scale.

We validated our approach by comparing the *B_ε_* (*T*) values obtained for ^4^He with those appearing in the literature, and we show in Fig. 2 the difference between the literature values (*B_ε,_*_lit_) and our results (*B_ε,_*_PIMC_, calculated with our path-integral approach but using the pair potential and pair polarizability of the original references). The error bars in Fig. 2 result from combining in quadrature our statistical uncertainty and the claimed uncertainty of the literature result. We compared our results with three literature sources: The first includes the work by Moszynski and coworkers wherein the theory was developed [16, 17]. In this case, we observed mutual agreement between our results and theirs, although we noticed a small systematic difference. The second source is the paper by Rizzo *et al.* [18]. These authors did not perform a rigorous analysis of the final uncertainty, but they stated their expectation that the results were converged to better than 1% and possibly within 0.1%. We found that our path-integral results were consistent with theirs assuming their worst case, especially at the highest temperatures. In fact, we managed to reproduce the results of Ref. [18] using the wavefunction approach, and we were able to check that the results given there were indeed converged only to 1% and not 0.1%, due to an insufficient number of angular momenta considered. Finally, we compared our results with the classical values reported by Cencek *et al.* [20]; in this case, the agreement was essentially perfect.

### 5.2 Estimate of the Uncertainty

Apart from the statistical uncertainty of the Monte Carlo method, which can be reduced arbitrarily by performing long enough simulations, and the uncertainty due to the convergence in the number of beads, which can also be reduced arbitrarily by using a large enough Trotter index *P*, there are two sources of uncertainty in the values of the second dielectric virial coefficient. These come from the uncertainty in the pair potential *V*_2_(*r*) and the uncertainty in the interaction-induced polarizability ∆*α*_2_(*r*).

We calculated *B_ε_* (*T*) for helium with the most accurate potential and polarizability currently available, namely the pair potential developed by Przybytek *et al.* [19] and the interaction-induced polarizability developed by Cencek *et al.* [20]. In both cases, the authors provided estimates of the uncertainty, and we evaluated the overall uncertainty of the second dielectric virial as follows.

Analogous to our previous work [15, 27, 28], we generated the “most repulsive” and the “most attractive” pair potentials, defined as *V*_±_(*r*) = *V*_2_(*r*) ± *δV*_2_(*r*), and evaluated the second dielectric virial in both cases using the given ∆*α*_2_(*r*). The absolute difference between the resulting *B_ε_* (*T*) values provides an estimate of the uncertainty due to the potential. We also evaluated the second dielectric virials using *V*_2_(*r*) with the perturbed interaction polarizabilities ∆*α*_±_(*r*) = ∆*α*_2_(*r*) ± *δ* ∆*α*_2_(*r*), obtaining an estimate of the uncertainty of *B_ε_* (*T*) due to the uncertainty of the polarizability by taking the absolute difference of the *B_ε_* obtained in the two cases. The overall uncertainty was estimated by the sum in quadrature of these two uncertainties. While neither Ref. [19] nor Ref. [20] assigned a rigorous statistical meaning to the “uncertainty” reported, in our judgment it is reasonable to treat them as expanded uncertainties with coverage factor *k* = 2, approximately corresponding to a 95% confidence interval. We will use the same expanded uncertainty for our uncertainty analysis in this paper.

By far the largest contribution to the uncertainty comes from the uncertainty in the interaction-induced polarizability ∆*α*_2_(*r*), which contributes an uncertainty in the second dielectric virial coefficient that is two to three orders of magnitude larger than the contribution due to the uncertainty of the pair potential. We performed Monte Carlo simulations using a large enough number of steps to make the statistical uncertainty much smaller (at least one order of magnitude) than the systematic uncertainty due to ∆*α*_2_(*r*). Nevertheless, we summed in quadrature also the statistical uncertainty to obtain the overall uncertainty of *B_ε_* (*T*).

For the refractivity virial coefficient *B*_R_, a rigorous uncertainty estimate is not possible, because no uncertainty estimate is available for the interaction Cauchy moment, ∆*S*(−4) (this is also the case for neon and argon). However, as will be apparent in subsequent sections, the relative difference between *B_ε_* and *B*_R_ at optical frequencies is small for most conditions (on the order of a few percent); a conservative estimate might add an additional uncertainty contribution of 10% of the magnitude of the correction.

### 5.3 **Numerical Values of** *B_ε_* (*T*) **for Helium**

The values of the second dielectric virial coefficient of ^4^He are reported in Table 1. The need to include quantum effects for this light atom is apparent by the discrepancy between quantum and classical values below 500 K. Table 1 also includes *B_ε_* for the rare isotope ^3^He, which finds use in cryogenics. As expected, the deviation from classical behavior is somewhat larger for the lighter ^3^He.

Since ∆*α*_2_(*r*) of helium is negative [31], the values of *B_ε_* in Table 1 are generally negative, with the exception of ^3^He at the lowest temperature. As Eq. (5) shows, the second dielectric virial coefficient is given by the sum of two terms, which, in the case of ^4^He (*I* = 0), have the same (negative) sign. However, ^3^He is a fermion (*I* = 1*/*2), and in this case the two contributions have opposite signs. At very low temperatures, the second term dominates in magnitude, resulting in a positive value of *B_ε_* for ^3^He.

**Table 1:** Values of the second dielectric and refractivity virial coefficients of ^4^He and ^3^He, together with the overall expanded (*k* = 2) uncertainty of *B_ε_* , *U* (*B_ε_* ), in units of 10^−3^ cm^6^ mol^−2^, and the dispersion correction $B_{R}^{\left(2\right)}$ defined in Eq. (3). These results were obtained with the pair potential of Ref. [19], the interaction-induced polarizability of Ref. [20], and the Cauchy moment of Refs. [11, 31]. In this and subsequent tables, values of the dispersion correction $B_{R}^{\left(2\right)}$ correspond to those to be used in Eq. (3) when the angular frequency *ω* is in atomic units; our example wavelength of 632.99 nm corresponds to *ω* = 0.071 981 a.u.

Temperature (K)	*B_ε_* (*T*), ^4^He	*B_ε_* (*T*), ^3^He	*U* (*B_ε_* )	*B_ε_* (*T*)(classical)	$B_{R}^{\left(2\right)}$, ^4^He	*B*__R__, ^4^He (632.99 nm)
1	-5.1	0.44	0.4	-172491	-120.0	-5.8
2	-2.3	-0.21	0.3	-913	-79.6	-2.7
3	-1.9	-0.68	0.2	-160	-70.6	-2.3
4	-2.0	-1.1	0.2	-65.7	-67.6	-2.3
5	-2.3	-1.5	0.2	-38.2	-66.5	-2.7
7	-3.0	-2.2	0.2	-20.6	-66.6	-3.4
10	-3.9	-3.3	0.2	-13.6	-69.0	-4.3
15	-5.6	-5.1	0.2	-11.1	-74.4	-6.0
20	-7.1	-6.7	0.2	-11.1	-80.2	-7.6
30	-10.2	-9.8	0.2	-12.7	-91.4	-10.7
40	-12.9	-12.5	0.2	-14.8	-101.7	-13.4
50	-15.3	-15.0	0.2	-17.0	-111.2	-15.9
75	-20.9	-20.6	0.2	-22.0	-132.0	-21.6
100	-25.8	-25.5	0.2	-26.7	-150.0	-26.5
125	-30.2	-29.9	0.2	-30.9	-166.0	-31.0
150	-34.2	-34.0	0.2	-34.9	-180.4	-35.1
175	-37.9	-37.7	0.2	-38.5	-193.6	-38.9
200	-41.4	-41.2	0.2	-42.0	-205.9	-42.4
250	-47.9	-47.7	0.2	-48.4	-228.1	-49.1
273.16	-50.7	-50.5	0.2	-51.1	-237.5	-51.9
300	-53.7	-53.6	0.2	-54.2	-247.9	-55.0
350	-59.1	-59.0	0.2	-59.5	-265.9	-60.5
400	-64.1	-64.0	0.2	-64.5	-282.4	-65.6
450	-68.9	-68.7	0.2	-69.2	-297.6	-70.4
500	-73:3	-73.2	0.2	-73.6	-311.9	-74.9
600	-81.5	-81.4	0.2	-81.8	-337.9	-83.3
700	-88.9	-88.8	0.2	-89.2	-361.2	-90.8
800	-95.8	-95.7	0.2	-96.0	-382.4	-97.8
900	-102.2	-102.1	0.2	-102.4	-401.8	-104.3
1000	-108.1	-108.0	0.2	-108.3	-419.8	-110.3
1500	-133.4	-133.4	0.2	-133.6	-494.1	-136.0
2000	-153.7	-153.6	0.2	-153.8	-551.5	-156.5

Inspection of the two contributions in Eq. (5) allows us to evaluate the temperature at which exchange becomes important. In the case of ^3^He, the exchange term becomes sizable (that is, of the order of 1% of the direct term) below 5 K and of the same magnitude at around 1.6 K. A similar situation is observed for ^4^He, although in this case the exchange term becomes of the order of 1% of the direct term below 4 K, and at the temperature of 1 K it is roughly 80% the value of the direct term. In this case, the increasing importance of the exchange term at low temperatures results in a maximum of *B_ε_* (*T*) around 3 K.

### 5.4 Second Refractivity Virial Coefficient of Helium

Table 1 also includes values of the dispersion correction $B_{R}^{\left(2\right)}$ for use in Eq. (3), allowing the calculation of the second refractivity virial coefficient *B*_R_. To be consistent with previous work [11, 18, 23], we tabulate this quantity corresponding to the use of atomic units for the angular frequency *ω* in Eq. (3). We also provide values of *B*_R_ at the wavelength of 632.99 nm, which is widely used in metrology. Our calculated values of $B_{R}^{\left(2\right)}$ agree with those given by Rizzo *et al.* [18], who used a different pair potential; our differences from their results are much less than 1% over most of the temperature range, with somewhat higher deviations below 20 K. Table 1 only presents refractivity information for ^4^He; results for ^3^He are given in the Supplemental Material.

### 5.5 Comparison of Helium Results with Experiment

**Fig. 3:**
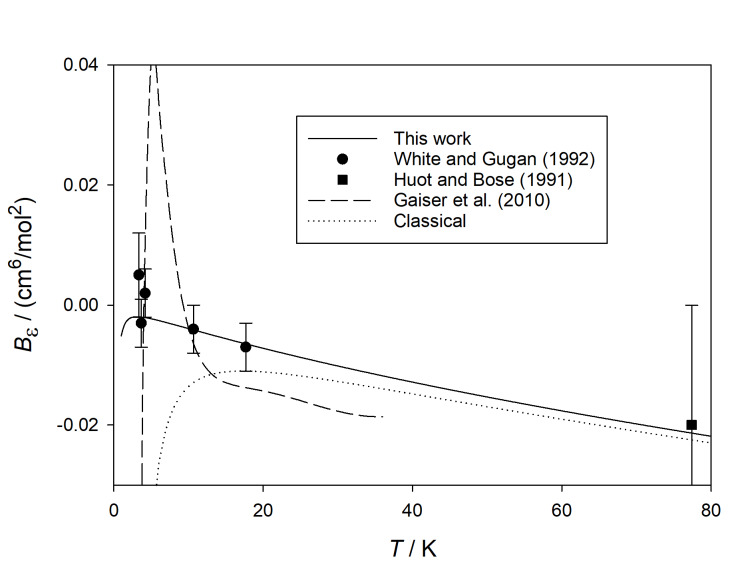
Comparison of calculated values of *B_ε_* (*T*) for ^4^He with those derived from experiment [44–46] at cryogenic temperatures.

Figure 3 shows the calculated values of *B_ε_* at cryogenic temperatures, along with available experimental data (with their reported standard uncertainties).

Gaiser *et al.* [44] reported a temperature correlation for (*B* − *b*), which they obtained from 3.7 K to 36 K using dielectric-constant gas thermometry. We converted these values to *B_ε_* = *A_ε_ b* using *B* computed (at the full quantum level) from the pair potential of Przybytek *et al.* [19]. Because (*B* − *b*) is orders of magnitude larger than *b*, it is not possible to obtain *B_ε_* with high accuracy using this method; the standard uncertainty in *b* varies from 0.02 cm^3^ mol^−1^ at the highest temperatures shown to 0.19 cm^3^ mol^−1^ at the lowest temperatures [47]. The deviation from our calculations shown on Fig. 3 for the correlation of Gaiser *et al.* [44] is within these uncertainties, although the shape of the deviation suggests that the function they chose to represent (*B* − *b*) may not quite have the right shape at the lowest temperatures. The older dielectric-constant gas thermometry experiments of Gugan and Michel [48] had too much uncertainty in (*B* − *b*) to allow a reliable estimate of *B_ε_* .

The expansion measurement of Huot and Bose at 77 K [45] is in good agreement within its relatively large uncertainty. A striking feature of Fig. 3 is the agreement with the expansion measurements performed by White and Gugan between 3 K and 18 K [46]. While our calculated results have much smaller uncertainties (see Table 1), White and Gugan deserve credit for making accurate measurements at challenging conditions.

Also shown on Fig. 3 is the result of the classical calculation of *B_ε_* . Not surprisingly, the error of this calculation becomes large below about 20 K. While the deviation of the classical calculation appears small on the graph above about 40 K, the error exceeds the expanded uncertainty of our calculation by roughly an order of magnitude in that range.

**Fig. 4:**
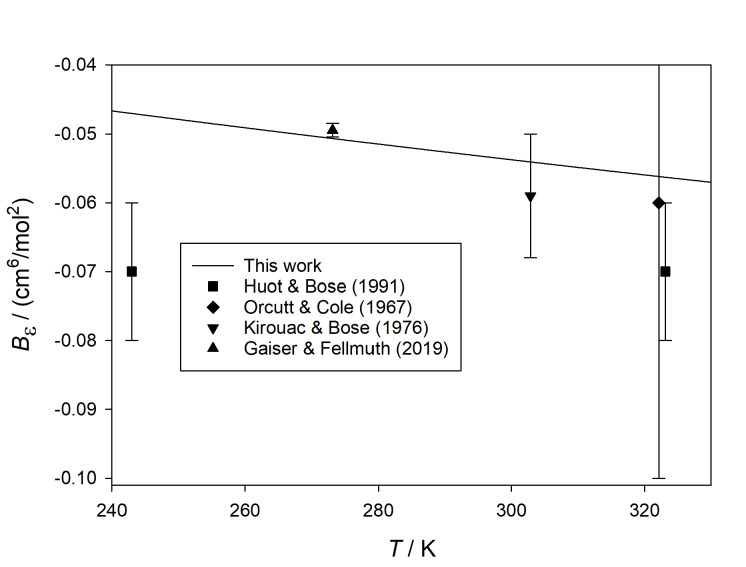
Comparison of calculated values of *B_ε_* (*T*) for ^4^He with those derived from experiment [42, 45, 49, 50] at higher temperatures.

Figure 4 shows our calculated values of *B_ε_* at higher temperatures, compared with several experimental sources [42, 45, 49, 50]. The older sources are somewhat scattered, but for the most part they are consistent with our results. Particularly noteworthy is the agreement with the recent work of Gaiser and Fellmuth [50], who reported (*B* − *b*) with small uncertainty at 273.1576 K from dielectric-constant gas thermometry. When converted to *B_ε_* with *B* calculated from the potential of Przybytek *et al.* [19], the resulting estimate of *B_ε_* is consistent with our results (which have about a factor of 10 smaller uncertainty).

For ^3^He, the only reported value of *B_ε_* comes from the capacitance measurements of Kerr and Sherman [51] near 3.8 K. Converted to our units, their result (after correcting an obvious sign error in the paper) is *B_ε_* = −0.030 cm^6^ mol^−2^, which is much too negative. This work was criticized by Gugan and Michel [48], who reanalyzed the experimental data but obtained a similar result. (We note that the value of *b* that Gugan and Michel attributed to Kerr and Sherman is too large in magnitude by a factor of 4/3, probably reflecting use of the molar mass of ^4^He rather than ^3^He.)

For the refractivity virial *B*_R_ of ^4^He, the only measurements we know of are those of Achtermann and coworkers [52, 53], who reported (−0.068 ± 0.010) cm^6^ mol^−2^ at both 303.15 K and 323.15 K at a wavelength of 632.99 nm. These lie slightly below our values for *B*_R_ (see Table 1), but they are consistent within their experimental uncertainty. We are not aware of any measurements of *B*_R_ for ^3^He.

## 6 Results for Neon

### 6.1 **Numerical Values of** *B_ε_* (*T*) **for Neon**

Natural neon exists in three stable isotopes. The amount of ^21^Ne is much less than 1%, while ^20^Ne is roughly 90.5% and ^22^Ne is roughly 9.2% [54]. In principle, a separate *B_ε_* could be computed for each isotopic pair and combined to yield an overall value of *B_ε_* for any isotopic composition, analogous to the procedure used to calculate the second virial coefficient *B* for a mixture. However, since the isotopic differences for neon are small, we simply tabulate the results for pure ^20^Ne and ^22^Ne.

The values of *B_ε_* (*T*) in Table 2 were obtained using the neon pair potential of Hellmann *et al.* [22] together with the pair polarizability of Hättig *et al.* [23]. Since neither paper reported uncertainties, we were unable to calculate the overall uncertainties of the second dielectric virials. It is reasonable to expect the relative uncertainties to be of the same order as those for argon, discussed below, since the *ab initio* calculations were performed at a similar level in both cases. However, Gaiser and Fellmuth [50] cited a private communication by Bich with an independent calculation of *B_ε_* at 273.16 K of −0.067 cm^6^ mol^−2^, which is significantly less negative than the −0.089 cm^6^ mol^−2^ we obtained based on the pair polarizability of Hättig *et al.* [23]. This suggests that there is a larger uncertainty in our knowledge of *B_ε_* for neon.

Reference [23] reported values of the second dielectric virial coefficient, as well as the second density virial, obtained with a pair potential described in the same paper. Although the authors stated that they used a semiclassical approximation, the values reported for both *B*(*T*) and *B_ε_* (*T*) came from purely classical calculations. In Ref. [23], the authors also used the method outlined in Sec. 2 to calculate the second dielectric virial coefficient. However, the values that they reported were not converged to the claimed precision due to an insufficient number of angular momenta used to perform the sum in Eq. (10), as we were able to verify by running our code implementing the same algorithm.

The data reported in Table 2 show that the quantum nature of the neon atom also has to be taken into account in this case; the temperature at which the deviation from a classical calculation becomes significant is around 200 K.

### 6.2 Second Refractivity Virial Coefficient of Neon

Table 2 includes values of $B_{R}^{\left(2\right)}$ and of *B*_R_ at the wavelength of 632.99 nm, again calculated at the fully quantum level. Table 2 only presents this information for ^20^Ne; results for ^22^Ne are presented in the Supplemental Material. Our values of $B_{R}^{\left(2\right)}$ in this case are somewhat more negative than those given by Hättig *et al.* [23], by about 2% at high temperatures and increasing to roughly 15% at 40 K. It is not clear whether this is the result of their different pair potential or some other difference in the calculations.

**Table 2:** Values of the second dielectric and refractivity virial coefficients of ^20^Ne and ^22^Ne in units of cm^6^ mol^−2^, along with the dispersion correction $B_{R}^{\left(2\right)}$ defined in Eq. (3). These results were obtained with the pair potential of Ref. [22], together with the interaction-induced polarizability and Cauchy moment of Ref. [23]. No uncertainty was reported in the original papers, and so we could not assign any uncertainty to these values.

Temperature (K)	*B_ε_* (*T*), ^20^Ne	*B_ε_* (*T*), ^22^Ne	*B_ε_* (*T*)(classical)	$B_{R}^{\left(2\right)}$, ^20^Ne	*B*__R__, ^20^Ne (632.99 nm)
4	-47.3	-55.1	-605	-378.9	-49.3
5	-12.5	-14.0	-80.9	-100.5	-13.0
7	-2.58	-2.79	-8.28	-21.2	-2.69
10	-0.744	-0.784	-1.51	-6.30	-0.777
15	-0.264	-0.273	-0.399	-2.35	-0.276
20	-0.152	-0.156	-0.203	-1.42	-0.159
30	-0.0879	-0.0882	-0.103	-0.85	-0.091
40	-0.0673	-0.0680	-0.0756	-0.67	-0.071
50	-0.0596	-0.0600	-0.0648	-0.59	-0.063
75	-0.0553	-0.0555	-0.0579	-0.51	-0.058
100	-0.0575	-0.0576	-0.0592	-0.50	-0.060
125	-0.0615	-0.0616	-0.0627	-0.50	-0.064
150	-0.0660	-0.0661	-0.0670	-0.51	-0.069
175	-0.0708	-0.0709	-0.0716	-0.52	-0.073
200	-0.0755	-0.0756	-0.0762	-0.54	-0.078
250	-0.0848	-0.0849	-0.0854	-0.57	-0.088
273.16	-0.0890	-0.0890	-0.0895	-0.58	-0.092
300	-0.0936	-0.0937	-0.0941	-0.60	-0.097
350	-0.102	-0.102	-0.102	-0.63	-0.105
400	-0.110	-0.110	-0.110	-0.65	-0.113
450	-0.117	-0.117	-0.118	-0.68	-0.121
500	-0.125	-0.125	-0.125	-0.70	-0.128
600	-0.138	-0.138	-0.138	-0.75	-0.142
700	-0.150	-0.150	-0.150	-0.79	-0.154
-0.800	-0.162	-0.162	-0.162	-0.83	-0.166
900	-0.172	-0.173	-0.173	-0.86	-0.177
1000	-0.183	-0.183	-0.183	-0.89	-0.187
1500	-0.226	-0.226	-0.226	-1.03	-0.231
2000	-0.261	-0.262	-0.261	-1.14	-0.267

### 6.3 Comparison with Experiment for Neon

**Fig. 5:**
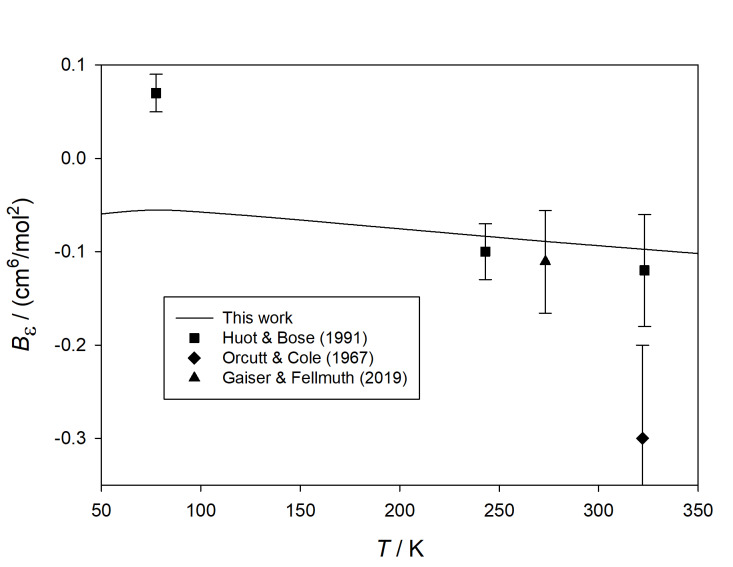
Comparison of calculated values of *B_ε_* (*T*) for neon with those derived from experiment [42, 45, 50].

In Fig. 5, our results for *B_ε_* (*T*) for neon are compared with experimental results [42, 45, 50]. We again achieved good agreement with the result derived by Gaiser and Fellmuth [50] from measuring (*B* − *b*) near 273 K, although their uncertainty is larger in this case because *B* for neon [55] is not known as accurately as for helium.

**Fig. 6:**
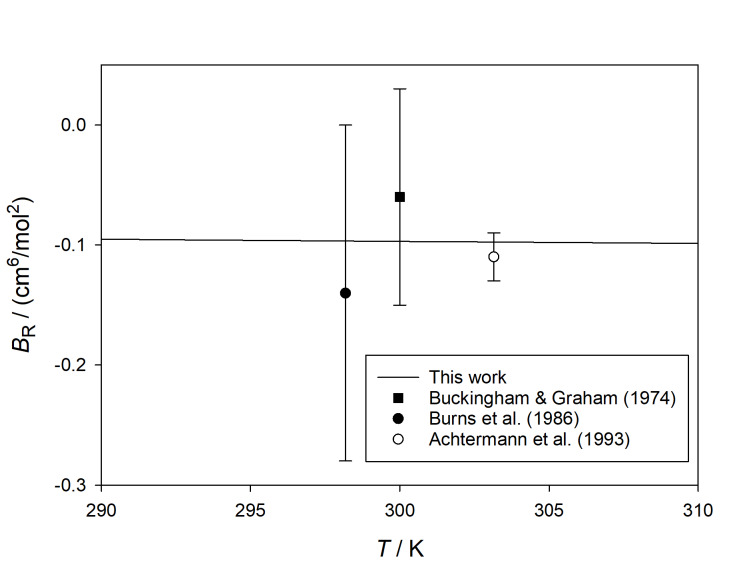
Comparison of calculated values of *B*__R__(*T*) for neon at a wavelength of 632.99 nm with those derived from experiment [53, 56, 57].

Three studies have reported *B*_R_ for neon [53, 56, 57]. While Burns *et al.* [57] reported data at multiple wavelengths, we limit our comparison to 632.99 nm, where all three studies reported results. Figure 6 shows that our calculations are consistent with all three results, including the most recent and precise datum of Achtermann *et al.* [53].

## 7 Results for Argon

### 7.1 **Numerical Values of** *B_ε_* (*T*) **for Argon**

We report our results for the second dielectric virial coefficient of the most abundant (~99.6%) argon isotope, ^40^Ar, in Table 3. For these calculations, we used the pair potential developed by Patkowski and Szalewicz [24] together with the pair polarizability of Vogel *et al.* [25]. Reference [25] reported an alternative pair potential, which gave indistinguishable results when we used it instead.

Comparison between the classical and quantum calculations of *B_ε_* (*T*) shows that argon behaves like a classical system at temperatures above roughly 100 K. In fact, our fully quantum values of *B_ε_* are in excellent agreement with those reported by Vogel *et al.* [25] and by Song and Luo [21], which were obtained with a semiclassical approach. We obtained expanded uncertainties with the same approach as for helium. While the uncertainty of the pair polarizability ∆*α*_2_(*r*) was not reported in Ref. [25], one of the authors communicated to us an estimated standard uncertainty of 5% [58].

The small influence of quantum effects on the dielectric virial of argon contrasts with the results reported by Rizzo *et al.* [18]. In Ref. [18], the comparison between classical results and quantum statistical calculations using the approach outlined in Sec. 2 indicated a 3% effect at 100 K, in contrast to the 0.4% difference between our quantum values and classical values at the same temperature. In fact, as we noted in Sec. 2.1, we were unable to obtain correct results using the approach starting from Eq. (6), despite using a large number of angular momenta or a very dense grid of wavevectors to account for the resonances in the scattering of ^40^Ar atoms. However, when we resorted to the alternative diagonalization method, the values of *B_ε_* (*T*) for *T* = 100 K using the same potential and polarizability as Ref. [18] were 2.253 cm^6^ mol^−2^ and 2.258 cm^6^ mol^−2^ in the quantum and classical case, respectively. PIMC calculations at the same conditions agree very well with the results from diagonalization. These values should be compared with the result of Rizzo *et al.* [18], *i.e.*, 2.23 cm^6^ mol^−2^ using a classical approach and 2.16 cm^6^ mol^−2^ using a quantum approach based on Eq. (5) and Eq. (6), which we believe is missing something in the case of ^40^Ar.

### 7.2 Second Refractivity Virial Coefficient of Argon

Table 3 includes calculated values of $B_{R}^{\left(2\right)}$ for argon, and *B*_R_ computed at 632.99 nm from Eq. (3). Our values of $B_{R}^{\left(2\right)}$ are systematically less positive (by around 8%) compared to the values calculated classically by Koch *et al.* [11]. Our classical calculations show that quantum effects on $B_{R}^{\left(2\right)}$ are less than 1% in the temperature range reported by Koch *et al.*, so the reason for the discrepancy is unclear.

**Table 3:** Values of the second dielectric and refractivity virial coefficients of ^40^Ar and the expanded (*k* = 2) uncertainty of *B_ε_* , *U* (*B_ε_* ), in units of cm^6^ mol^−2^, along with the dispersion correction $B_{R}^{\left(2\right)}$ defined in Eq. (3). These results were obtained with the pair potential of Ref. [24], the pair polarizability of Ref. [25], and the Cauchy moment of Refs. [11, 31]. In the calculation of the uncertainty, a 5% standard uncertainty of the pair polarizability was assumed [58].

Temperature (K)	*B_ε_* (*T*)	*U* (*B_ε_* )	*B_ε_* (*T*)(classical)	$B_{R}^{\left(2\right)}$	*B*__R__(632.99 nm)
50	6.17	0.35	6.25	-4.72	6.14
75	3.53	0.20	3.54	3.31	3.54
100	2.75	0.16	2.76	4.83	2.78
125	2.39	0.14	2.40	5.25	2.42
150	2.18	0.13	2.18	5.35	2.21
175	2.04	0.13	2.04	5.33	2.07
200	1.94	0.12	1.94	5.25	1.97
250	1.80	0.12	1.80	5.03	1.83
273.16	1.76	0.11	1.76	4.92	1.78
300	1.71	0.11	1.71	4.79	1.73
350	1.64	0.11	1.64	4.54	1.66
400	1.58	0.11	1.58	4.30	1.60
450	1.53	0.11	1.53	4.07	1.55
500	1.49	0.11	1.49	3.85	1.51
600	1.42	0.11	1.42	3.43	1.43
700	1.36	0.11	1.36	3.04	1.37
800	1.30	0.11	1.30	2.68	1.32
900	1.26	0.12	1.25	2.35	1.27
1000	1.21	0.12	1.21	2.03	1.22
1500	1.03	0.12	1.03	0.71	1.04
2000	0.90	0.13	0.89	−0.33	0.89

### 7.3 Comparison with Experiment for Argon

**Fig. 7:**
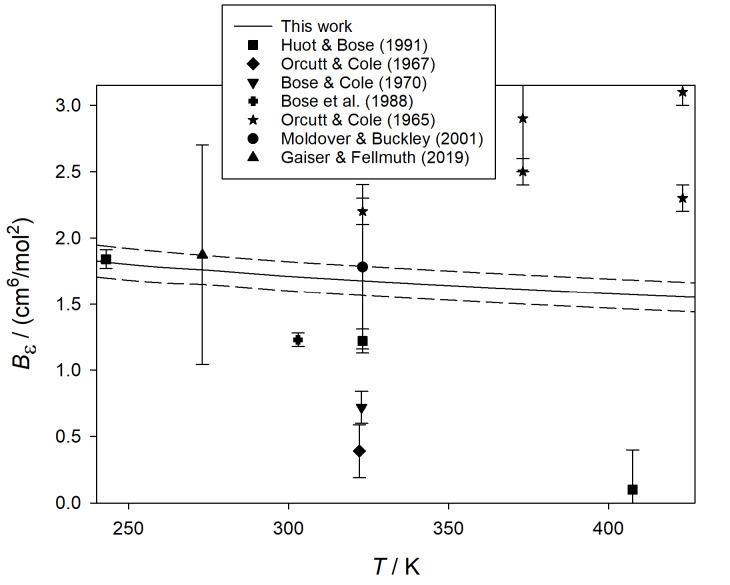
Comparison of calculated values of *B_ε_* (*T*) for argon with those derived from experiment [42, 45, 50, 59–62]. Dashed lines indicate expanded (*k* = 2) uncertainty of calculated values.

In Fig. 7, our results for *B_ε_* (*T*) for argon are compared with the available data [42, 45, 50, 59–62]. In addition to expansion measurements, we included the older direct dielectric measurements of Orcutt and Cole [59], because they extend to higher temperatures, and a more recent direct measurement of Moldover and Buckley [62] made with a cross capacitor. There is much scatter in the experimental values, especially at high temperatures, and some of the reported experimental uncertainties must be overly optimistic.

**Fig. 8:**
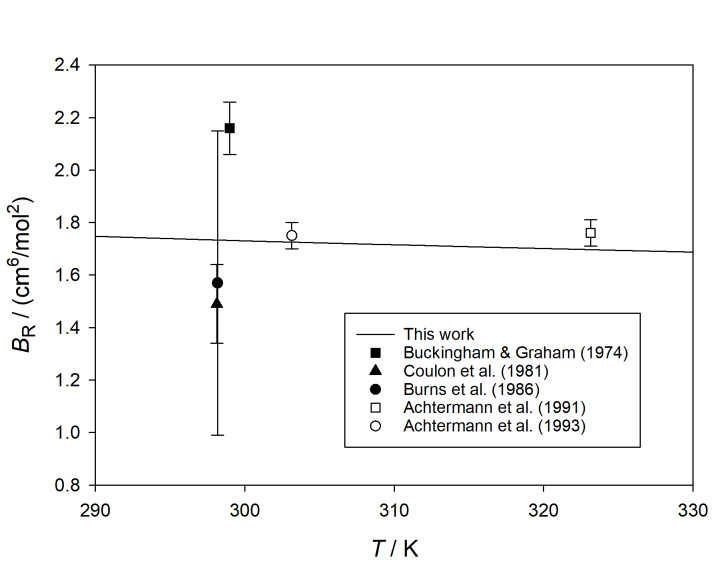
Comparison of calculated values of *B*__R__(*T*) for argon at a wavelength of 632.99 nm with those derived from experiment [52, 53, 56, 57, 63].

Five studies have reported *B*_R_ for argon [52, 53, 56, 57, 63]. While two of these [57, 63] reported data at multiple wavelengths, we limit our comparison to 632.99 nm, where all studies reported results. Figure 8 shows that we again obtained good agreement with the more recent data of Achtermann and coworkers [52, 53], and were within the scatter of the older data.

## 8 Conclusions

We developed a numerical procedure to evaluate the second dielectric and refractivity virial coefficients of atomic gases using the path-integral formulation of quantum statistical mechanics. This approach enabled us to take into account both diffraction and quantum statistical effects with no uncontrolled approximations. The procedure was validated by demonstrating that it gives results matching those from the traditional, and generally more computer-intensive, phase-shift approach.

We applied our method to the calculation of the second dielectric and refractivity virial coefficients of ^4^He, ^3^He, neon, and argon using state-of-the-art pair potentials, interaction-induced polarizabilities, and interaction Cauchy moments. Additionally, for helium and argon, we evaluated the uncertainty of the dielectric virials due to the uncertainties in the potential and polarizability, finding that the contribution of the latter is much larger than that of the former. Our results are consistent with the (limited and scattered) experimental data available, but the calculated results have smaller uncertainties, especially for helium. Similar agreement was obtained with experiment for the second refractivity virial coefficients.

For helium, independent sources of both the pair potential and the pair polarizability are in good agreement, and we can be confident that neither quantity is introducing extra systematic uncertainty. For argon, it would be useful to have independent confirmation of the interaction polarizabilities of Vogel *et al.* [25], which have 5% uncertainty but yield *B_ε_* inconsistent with the older results of Rizzo *et al.* [18] (even after accounting for the inaccuracy in the quantum calculations of Ref. [18] discussed in Sec. 7.1). For neon, the situation is worse; the only published pair polarizability is that of Hättig *et al.* [23], for which the uncertainty is unknown. Furthermore, the unpublished personal communication of *B_ε_* at 273.16 K cited by Gaiser and Fellmuth [50] disagrees with the *B_ε_* in Table 2 by roughly 25%, implying that either the pair polarizability of Hättig *et al.* or the one used in the unpublished work is significantly in error. For use of neon in metrology based on dielectric or refractive measurements, an independent calculation of the interaction polarizability of neon, with reliable uncertainty estimates, would be highly desirable.

Because metrology will require these quantities at temperatures not listed in Tables 1–3, we have deposited supplemental data files in which the quantities computed in this work are given at intervals of 1 K, with finer intervals below 10 K for ^3^He and ^4^He [64]. For metrological applications, we recommend interpolation in these files, rather than attempting to interpolate the values in the sparser Tables 1–3.

The inclusion of quantum effects in the calculations is important for quantitative accuracy, especially for helium. Classical calculations begin to deviate from the rigorous result by more than 1% below roughly 300 K for helium, 175 K for neon, and 60 K for argon.

While this paper was in preparation, Song and Luo [21] published values of *B_ε_* for the same species we considered here, calculated at a semiclassical level including second-order quantum corrections. They used the same pair potentials and ∆*α*_2_(*r*) that we used in this work. The agreement with our fully quantum results is excellent down to roughly 20 K for ^4^He, 30 K for ^3^He, and at all temperatures tabulated by Song and Luo for the other two gases (down to 25 K for neon and 83.806 K for argon).

Analogous to what has been done for the density virial coefficients [15, 26], our approach can be generalized in a direct way to higher-order dielectric virial coefficients, and we are working on that extension. However, rigorous calculation of *C_ε_* requires the nonadditive three-body interaction polarizability, and to the best of our knowledge no *ab initio* calculations of that quantity have been published.

## Supplemental Materials

See Ref. [64] for fully quantum values of *B_ε_*, $B_{R}^{\left(2\right)}$, and *B*_R_ at a wavelength of 633.99 nm at 1 K intervals (with finer intervals for helium below 10 K). Expanded (*k* = 2) uncertainties for *B_ε_* are given for ^3^He, ^4^He, and ^40^Ar. Classically calculated values of *B_ε_* are also tabulated for helium, neon, and argon.
